# Hard ticks (Ixodida: Ixodidae) in the Colombian Caribbean harbor the Jingmen tick virus: an emerging arbovirus of public health concern

**DOI:** 10.1186/s13071-024-06362-x

**Published:** 2024-06-25

**Authors:** Yesica López, Richard Thomas, Sebastián Muñoz-Leal, Yeimi López-Mejia, Ketty Galeano, Alejandra Garcia, Luis Romero, Daniel Echeverri-De la Hoz, Caty Martinez, Alfonso Calderón, Bertha Gastelbondo, Héctor Contreras, Gino Olivieri, Luis Rubiano, Luis Paternina, Richard Hoyos-López, Anggie Ortiz, Evelyn Garay, Maira Alemán-Santos, Ricardo Rivero, Jorge Miranda, Luis Florez, Jolaime Ballesteros, Verónica Contreras, Vaneza Tique, Pedro Fragoso, Camilo Guzman, German Arrieta, Salim Mattar

**Affiliations:** 1 Instituto de Investigaciones Biológicas del Trópico, Universidad de Córdoba, Córdoba, Colombia; 2 Departamento de Ciencia Animal, Facultad de Ciencias Veterinarias, Universidad de Concepción, Chillán, Chile; 3 Grupo de Investigación Parasitología y Agroecología Milenio, Universidad Popular del Cesar, Valledupar Cesar, Colombia; 4 Universidad de Sucre, Investigaciones Biomédicas, Sucre, Colombia; 5 Paul G. Allen School for Global Health, Washington State University, Pullman, WA USA; 6 Grupo de Investigaciones Microbiológicas y Biomédicas de Córdoba-GIMBIC, Universidad de Córdoba, Montería, Colombia; 7 Grupo de Salud Pública y Auditoría en Salud, Corporación Universitaria del Caribe- CECAR, Sincelejo, Colombia

**Keywords:** Jingmen tick virus, Virus, Tick-borne disease, Next-generation sequencing, *Rhipicephalus microplus*, *Dermacentor nitens*, *Amblyomma dissimile*

## Abstract

**Background:**

Ticks are obligate hematophagous ectoparasites involved in transmitting viruses of public health importance. The objective of this work was to identify the Jingmen tick virus in hard ticks from the Colombian Caribbean, an arbovirus of importance for public health.

**Methods:**

Ticks were collected in rural areas of Córdoba and Cesar, Colombia. Taxonomic identification of ticks was carried out, and pools of 13 individuals were formed. RNA extraction was performed. Library preparation was performed with the MGIEasy kit, and next-generation sequencing (NGS) with MGI equipment. Bioinformatic analyses and taxonomic assignments were performed using the Galaxy platform, and phylogenetic analyses were done using IQ-TREE2.

**Results:**

A total of 766 ticks were collected, of which 87.33% (669/766) were *Rhipicephalus microplus*, 5.4% (42/766) *Dermacentor nitens*, 4.2% (32/766) *Rhipicephalus linnaei*, and 3.0% (23/766) *Amblyomma dissimile*. Complete and partial segments 1, 2, 3, and 4 of Jingmen tick virus (JMTV) were detected in the metatranscriptome of the species *R. microplus*, *D. nitens*, and *A. dissimile*. The JMTVs detected are phylogenetically related to JMTVs detected in *Aedes albopictus* in France, JMTVs detected in *R. microplus* in Trinidad and Tobago, JMTVs in *R. microplus* and *A. variegatum* in the French Antilles, and JMTVs detected in *R. microplus* in Colombia. Interestingly, our sequences clustered closely with JMTV detected in humans from Kosovo.

**Conclusions:**

JMTV was detected in *R. microplus*, *D. nitens*, and *A. dissimile*. JMTV could pose a risk to humans. Therefore, it is vital to establish epidemiological surveillance measures to better understand the possible role of JMTV in tropical diseases.

**Graphical Abstract:**

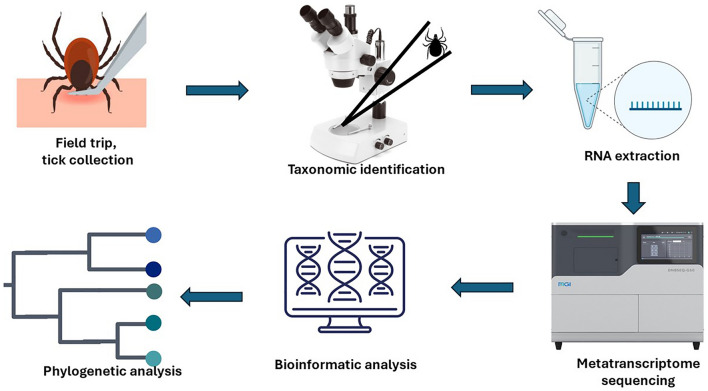

## Background

Tick-borne infections constitute a significant portion of vector-borne diseases, can generate a considerable disease burden, and present an imminent threat to public health. Ticks act as biological vectors for a wide range of microorganisms, including RNA viruses that can be transmitted to humans and animals [1].

Some examples of pathogenic viruses transmitted by ticks to humans or animals are Crimean−Congo hemorrhagic fever virus (CCHF), Nairobi sheep disease virus, tick-borne encephalitis virus, Omsk hemorrhagic fever, Kyasanur forest disease virus, Powassan virus, Alkhurma hemorrhagic fever virus, severe fever with thrombocytopenia virus, Heartland virus, Colorado fever virus, Bourbon virus, and African swine fever virus (ASF) [2, 3]. In addition, of these pathogens, Jingmen tick virus (JMTV) associated with disease in humans has recently been described [4].

JMTV was first detected in 2010 from China in *Rhipicephalus microplus* [5]. The virus belongs to the Jingmen virus group (JMV), and although it remains unclassified by the International Committee on Taxonomy of Viruses (ICTV), it is related to the Flavivirus genus of the *Flaviviridae* family [5]. The JMV group are single-stranded RNAs in the positive sense (ssRNA+), and unlike typical flaviviruses, it has a segmented genome [6].

The JMTV genome comprises four segments: S1, S2, S3, and S4. The S1 and S3 encode nonstructural proteins (NSPs), while the S2 and S4 encode structural proteins (VPs) [7]. S1 encodes NSP1, an RNA-dependent RNA polymerase homologous to the NS5 protein of flaviviruses; S2 presents an open reading frame (ORF) that encodes a glycoprotein (VP1), S3 encodes NSP2, homologous to the flavivirus NS3-NSP2 complex; S4 presents two ORFs to encodes two proteins, the capsid protein (VP2) and the membrane protein (VP3) [5]. Although segments S1 and S3 are closely related to the NSPs genes (NS3 and NS5) of flaviviruses, the remaining two segments (S2 and S4) are unique to these viruses and lack homologs [5].

JMTV has been identified in several regions of China in ticks and humans [4, 5, 8]. Additionally, its presence has been reported in ticks from various countries including Poland, Georgia, France, French Antilles, Laos People’s Democratic Republic, Brazil, Kenya, Turkey, Japan, Romania, Trinidad and Tobago, and Colombia [9–17], as well as mosquitoes (*Aedes albopictus*) in Italy [18]. JMTV has also been detected in vertebrates, such as the red colobus monkey (*Procolobus rufomitratus*) in Uganda [19], bats in Cambodia [9], cattle in Brazil [10], and rodents in China [20].

Remarkably, in Kosovo, JMTV was detected using NGS in human sera positive to CCHF [21]. Additional reports include human cases with symptoms such as fever, headache, and a history of tick bites [4, 22]. However, other clinical manifestations including lymphadenopathy, painful eschar, and itching at the tick bite site, have also been observed. These findings suggest a potential pathogenic role of this emerging tick-borne virus in humans [4].

The spread of viruses transmitted by ticks can go unnoticed until symptomatic cases arise or local epidemics affecting humans or domestic animals occur. Establishing effective surveillance systems is essential for the prompt detection of circulating pathogenic viruses that might pose a threat to public health [6].

The objective of this work was to identify the Jingmen tick virus in hard ticks from the Colombian Caribbean, an arbovirus of importance for public health.

## Methods

### Collection and taxonomic identification of ticks

**Fig. 1:**
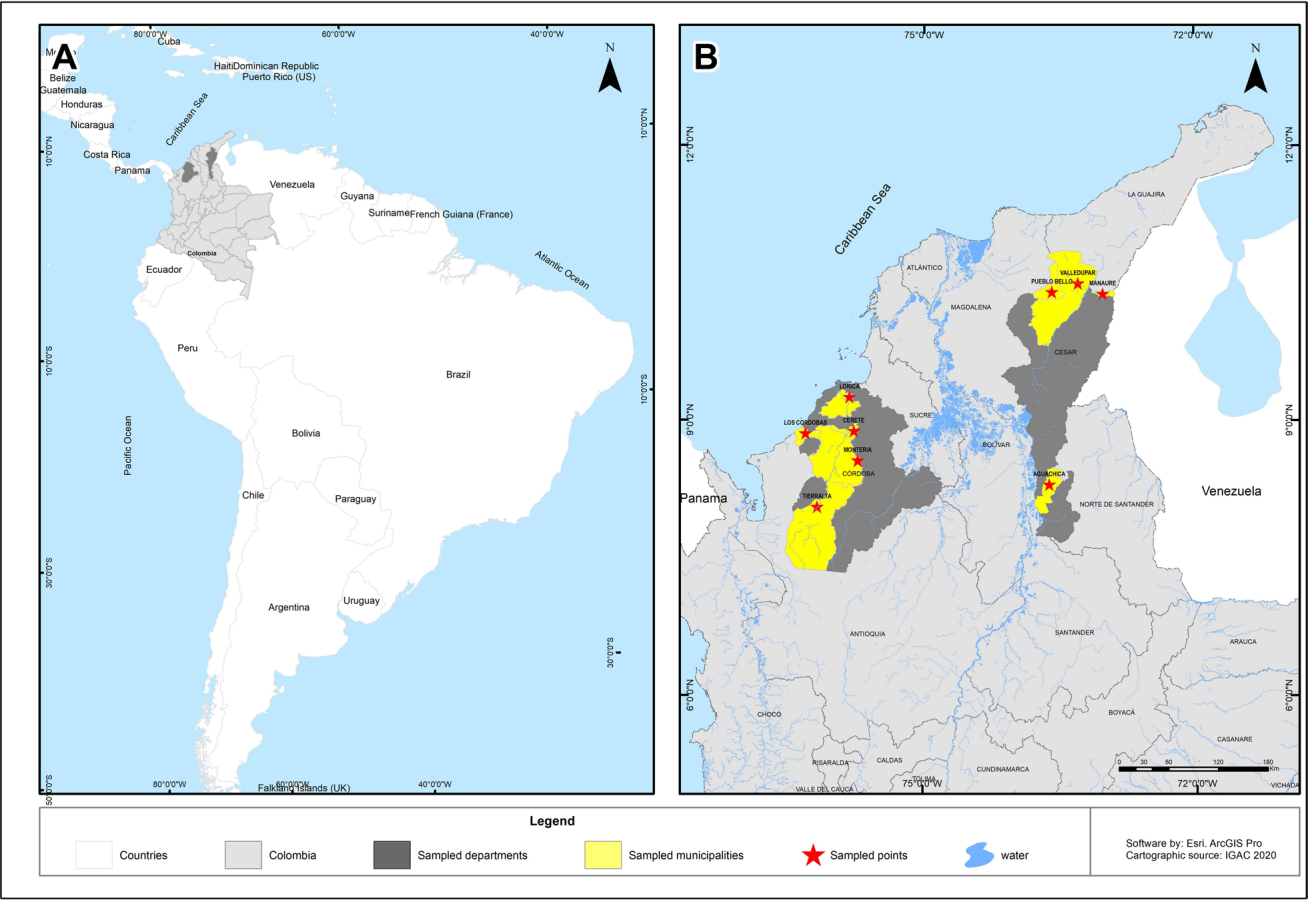
Geographic location of tick capture. **A** Map of South America showing the location of the departments of Córdoba and Cesar within Colombia. **B** Map of Colombia showing the sampled municipalities in the departments of Córdoba and Cesar

Between 2022 and 2023, field trips were carried out at different locations in the departments of Córdoba and Cesar to collect ticks (Fig. 1). The ticks were collected directly from wild and domestic animals such as cattle, horses, sheep, canines, snakes, and iguanas. Ticks were transported in liquid nitrogen and stored at −80 °C. The specimens were identified taxonomically [23] and grouped by genus, sex, and stage, with a maximum of 12 individuals.

### RNA extraction, library preparation, and sequencing

The tick pools were macerated in 600 μL PBS, and 200 μL of the supernatant was filtered with a 22-μm filter. Subsequently, DNA was removed from the samples using Promega’s RQ1 RNase-Free DNase reagent according to the manufacturer’s instructions. Extraction was carried out with a GeneJET RNA purification kit, following the manufacturer’s instructions. To initiate the sequencing process, 13 pools of tick RNA were generated taking into account genus, species, stage, and geographic location. RNA concentration and integrity number (RIN) was measured by fluorometry using the Qubit™ with RNA Quantification broad range (BR) and RNA IQ Assay Kit (Thermo Fisher Scientific), respectively. For the preparation of libraries, an input of 250–500 ng of total RNA was determined. The samples were processed with the MGIEasy Fast RNA library preparation set under the high-throughput DNA nanoball (DNB) sequencing methodology.

Initially, RNA was fragmented into products of approximately 250 nucleotides according to the FCL 150 paired-end (PE) platform. The first and second strands of DNA were then synthesized with random hexamer primers. The fragments were subjected to a process of end repair (ERAT), ligation of molecular barcodes, and amplification of products by polymerase chain reaction (PCR). The concentration of the library was obtained with the Qubit™ dsDNA Quantification Assay Kit, whereas the size of the fragments was determined with Fragment Analyzer™ (Agilent Technologies).

According to the concentration and size of the fragments, two pools with final mass of 1000 fmol/pool were obtained for circularization of DNAs and DNB synthesis (> 11 ng/μl). Finally, the cartridge, flow cell, and DNBs were loaded into the MGI-G50 machine to perform NGS of short reads (Shenzhen, China).

### Bioinformatic analysis

Low-quality sequences (Phred score < Q15), short reads (less than 15 bp), and adapter sequences were removed using fastp v.0.23.2 [24]. The genome sequences from different tick species analyzed were discarded through reference mapping using Bowtie2 v.2.5.0 [25]. De novo assembly was performed to obtain viral contigs or scaffolds using MEGAHIT v.1.2.9 [26]. Finally, the taxonomic assignment was performed with BLASTx 2.14.1 [27]. All these tools were accessed through the online platform Galaxy [28].

### Phylogenetic analysis

The obtained contigs were compared with the GenBank database using BLASTp [27]. Alignments were generated with MAFFT [29] with sequences downloaded from GenBank [30]. Phylogenetic reconstructions were performed in IQ-TREE v2.2.2.6 [31] with the maximum likelihood method [32]; the best-fit amino acid substitution models were obtained using ModelFinder, and the substitution models were chosen according to the Bayesian information criterion [33] and SH-like approximate likelihood ratio test for statistical support [34]. Trees were reconstructed using 1000 bootstraps visualized in iTOL v5 [35] and edited in Inkscape v.1.1 [36].

## Results

A total of 766 ticks were collected, of which 87.3% (669/766) were *R. microplus*, 5.4% (42/766) *Dermacentot nitens*, 4.2% (32/766) *Rhipicephalus linnaei*, and 3.0% (23/766) *Amblyomma dissimile*. In total, 120 pools were generated.

In the metatranscriptome of the species *R. microplus* and *D. nitens* collected in the Cesar Department, the complete genome of JMTV was detected. Additionally, complete and partial fragments of segments 1, 2, 3, and 4 that encode the proteins NSP1, VP1, NSP2, VP2, and VP3 of JMTV were detected in *R. microplus*, *D. nitens*, and *A. dissimile* collected in Córdoba and Cesar (Table 1).

**Table 1:** Groupings of JMTV-positive ticks by NGS

Code	Species	Sex/stage	Host	Department	Genetic segments of JMTV				
					NSP1	VP1	NSP2	VP2	VP3
6G	*A. dissimile*	♂	Iguana	Cesar	913aa	580aa	808aa	442aa	–
7G	*A. dissimile*	♀	Iguana	Cesar	–	–	–	–	254aa
9G	*D. nitens*	♀	Bovine	Cesar	888aa	657aa	808aa	442aa	254aa
10G	*D. nitens*	♂	Bovine	Cesar	913aa	752aa	808aa	538aa	254aa
11G	*R. microplus*	♀	Bovine	Cesar	563aa	752aa	639aa	460aa	254aa
12G	*R. microplus*	♂	Bovine	Cesar	913aa	752aa	808aa	531aa	254aa
13G	*R. microplus*	N	Bovine	Cesar	913aa	752aa	808aa	531aa	254aa
26G	*R. microplus*	♀	Bovine	Córdoba	561aa	624aa	–	538aa	–
29G	*R. microplus*	♂	Bovine	Córdoba	913aa	752aa	–	538aa	–
117G	*R. microplus*	N	Bovine	Córdoba	913aa	529aa	–	538aa	–

*aa* amino acid

**Fig. 2:**
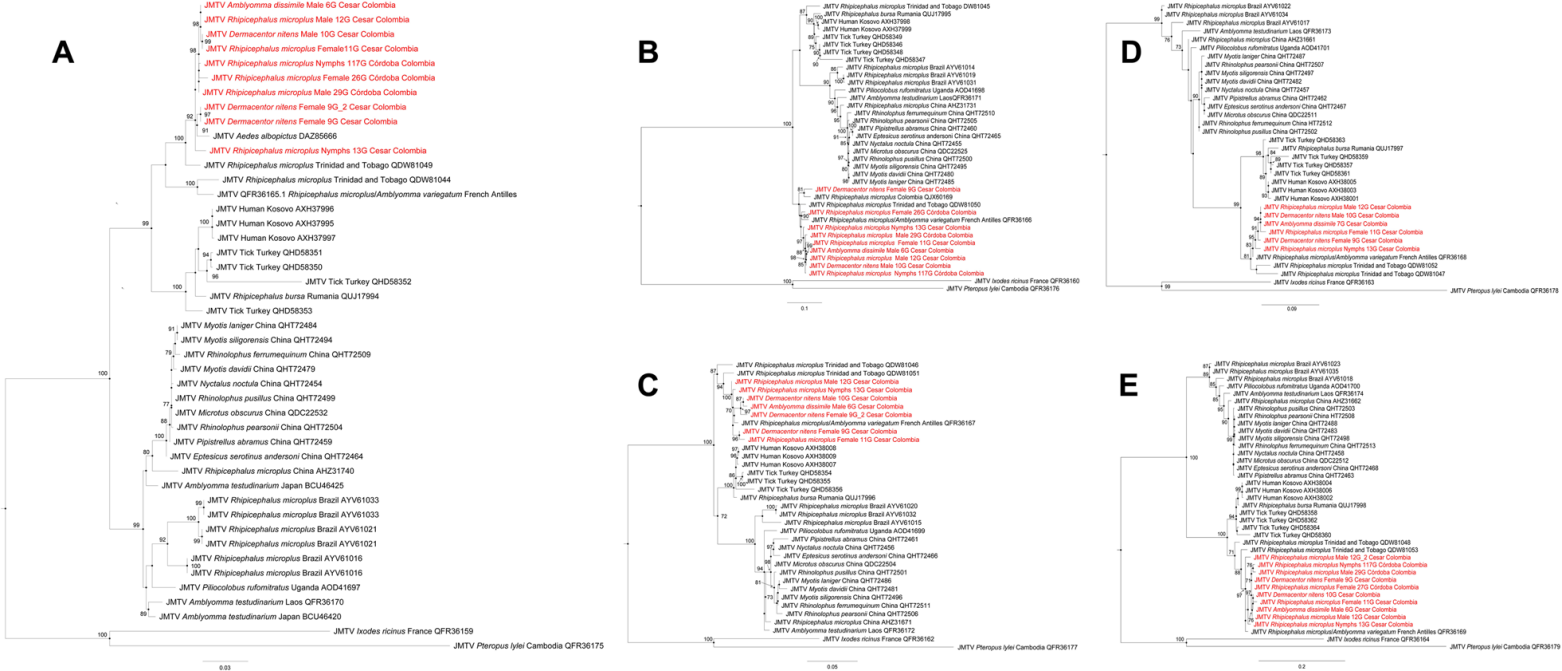
Phylogenetic reconstructions with amino acids of the four segments of JMTV. **A** Phylogenetic tree of the NSP1 protein built with 45 sequences (35 downloaded from GenBank and 10 own). **B** Phylogenetic tree of the VP1 protein built with 37 sequences (28 downloaded from GenBank and 9 own). **C** Phylogenetic tree of the NSP2 protein built with 35 sequences (28 downloaded from GenBank and 7 own). **D** VP2 phylogenetic tree built with 35 sequences (25 downloaded from GenBank and 10 own). **E** VP3 phylogenetic tree built with 39 sequences (33 downloaded from GenBank and 6 own). These five trees were rooted with JMTV (QFR36167) and JMTV (QFR36160). The sequences generated in this study are highlighted in red. Trees were constructed using the substitution models JTTDCMut + G4 for NSP1, FLU + F + G4 for VP1, FLU + G4 for VP2, JTT + G4 for NSP2, and VP3 protein

The JMTVs detected in this study are phylogenetically related to JMTVs detected in *A. albopictus* from France, JMTVs detected in *R. microplus* from Trinidad and Tobago, JMTVs detected in *R. microplus* and *A. variegatum* from the French Antilles, and JMTVs detected in Colombia. This relationship was consistent in all constructed trees (Fig. 2). Additionally, when performing BLASTp, the amino acid sequences presented a percentage of identity between 96.23% and 99.38% with the JMTV mentioned above [7, 11, 16].

Additionally, there is a phylogenetic relationship between the JMTVs detected in this study and those detected in humans in Kosovo, given that in trees A, D, and E the sequences of these groups are in the same cluster (Fig. 2, tree A, D, E).

## Discussion

JMTV was detected in *R. microplus* and *D. nitens* ticks collected from cattle, and *A. dissimile* collected from iguanas in the Colombian Caribbean (Table 1). This is the third JMTV study on *R. microplus* and the first virus report on *D. nitens* and *A. dissimile* from Colombia.

JMTV was first identified in *R. microplus* ticks in the Jingmen area of Hubei Province, China, in 2010 [5], then in 2012 in Uganda, JMTV was detected by NGS in the plasma of a red colobus monkey (*Procolobus rufomitratus*) [19]. Between 2013 and 2015, 12 human sera samples collected in Kosovo were analyzed, and patients with CCHF coinfected with JMTV were detected [21]. Between 2014 and 2016, in France, French Antilles, Laos People’s Democratic Republic, and Cambodia, JMTV was detected in *Ixodes ricinus*, *R. microplus*, *Amblyomma testudinarium*, and bats, respectively [9]. In 2017, JMTV was isolated in BME/CTVM23 cells. NGS sequenced the whole genome in *Amblyomma javanense* collected from Chinese pangolins [4]. That same year, human patients positive for JMV were reported in China. The infection was confirmed by RT-PCR assay in blood in 86 patients with fever, headache, and a history of tick bites. Serological assays showed seroconversion in 19 patients [22]. In Kenya, between 2013 and 2019, ticks of the *Amblyomma* and *Rhipicephalus* species were collected, and JMTV was detected by PCR and NGS [11].

Recently, in 2023, in China, JMTV was detected in *R. microplus* ticks through NGS (MGISEQ-2000), which PCR confirmed, and the complete genome was obtained with Sanger sequencing [8]. Furthermore, they detected JMTV in *I. ricinus* in Poland and Georgia by NGS (nanopore) and PCR [3].

In Latin America, there are a few reports of JMTV. In Brazil, between 2015 and 2016, complete and partial JMTV genomes were obtained by high-throughput sequencing in 67% (4/6) of tick pools and 14% (5/36) of bovine sera [10]. In Colombia, in the Department of Antioquía, between 2013 and 2018, several JMTV segments were detected by NGS in ticks of the genus *Rhiphicephalus* [16, 17]. Many of the JMTV reports with NGS carry out a confirmation with PCR; however, in this study, complete genomes were recovered. Thus, confirmation was unnecessary.

JMTV has a worldwide distribution and has been detected in six genera of ticks: *Rhipicephalus*, *Amblyomma*, *Dermacentor*, *Haemaphysalis*, *Hyalomma*, and *Ixodes*, and 26 species [7, 37]. This work provides two new species of ticks where JMTV was detected: *D. nitens* and *A. dissimile*. However, *R. linnaei* was negative despite previous positive reports for this tick species [5].

Interestingly, in the present work, JMTV was detected in *D. nitens* parasitizing cattle, which suggests transmission to cattle by these tick species, given that there are reports of this virus in cattle from Brazil [10]. This finding indicates a probable risk of transmission to different species of ticks, animals, and humans.

Phylogenetic analyses of five JMTV proteins showed that, in four of the trees obtained (Fig. 2, tree A, C–E), most of the viruses were grouped in two clusters, one of them associated with different species of bats and ticks and the other with humans and different species of ticks.

Interestingly, our sequences of the NSP1, VP2, and VP3 trees were placed in a clade closely related to the JMTVs detected in humans from Kosovo [21]. This is a finding that should alert the medical community in the tropical regions of Colombia.

Given the close linkage between the sole JMTV sequence identified in *A. albopictus* in Italy and the strain characterized in the current study (Fig. 2, tree A), it is necessary to carry out epidemiological surveillance of JMTV in mosquitoes from tropical areas of Colombia.

Unfortunately, for the phylogenetic analyses, only the VP1 of JMTV previously reported in Colombia could be included given the limited availability of data; however, it presents a high phylogenetic relationship with the JMTV detected in *D. nitens* from Cesar, reported in the present study.

It is necessary to develop serological tests with the exclusive segments of circulating JMTV to avoid cross-reactions with other endemic flaviviruses and generate tools to contribute to diagnosing febrile syndrome without an apparent focus.

## Conclusions

This is the first work in the Colombian Caribbean on ticks of importance in human and animal health. JMTV is one of the emerging viruses with a potential risk of causing epidemiological outbreaks because they are endemic globally. They are closely associated with arthropods and have caused sporadic cases of febrile illness in humans with a history of tick bites. These results are essential to establish surveillance measures to prevent possible outbreaks of these pathogens.

## Data Availability

Sequences are available in the Sequence Read Archive (SRA) with accession numbers SRR29433420, SRR29433419, SRR29433418, SRR29433417, SRR29433416, SRR29433415, SRR29433414, SRR29433413, SRR29433412, and SRR29433411.
